# Association of Plasma Amylin Concentration With Alzheimer Disease and Brain Structure in Older Adults

**DOI:** 10.1001/jamanetworkopen.2019.9826

**Published:** 2019-08-21

**Authors:** Haihao Zhu, Qiushan Tao, Ting Fang Alvin Ang, Joseph Massaro, Qini Gan, Saraf Salim, Rui-ying Zhu, Vijaya B. Kolachalama, Xiaoling Zhang, Sheral Devine, Sanford H. Auerbach, Charles DeCarli, Rhoda Au, Wei Qiao Qiu

**Affiliations:** 1 Department of Pharmacology & Experimental Therapeutics, Boston University School of Medicine, Boston, Massachusetts; 2 Department of Epidemiology, School of Public Health, Boston University School of Medicine, Boston, Massachusetts; 3 Framingham Heart Study, Boston University School of Medicine, Boston, Massachusetts; 4 Department of Psychiatry, Boston University School of Medicine, Boston, Massachusetts; 5 Department of Medicine, Boston University School of Medicine, Boston, Massachusetts; 6 Department of Neurology, Boston University School of Medicine, Boston, Massachusetts; 7 Alzheimer’s Disease Center, Boston University School of Medicine, Boston, Massachusetts; 8 Department of Anatomy & Neurobiology, Boston University School of Medicine, Boston, Massachusetts; 9 Alzheimer’s Disease Center, University of California Davis Medical Center, Sacramento

## Abstract

**Importance:**

Preclinical studies suggest that amylin has a U-shaped dose-response association with risk of Alzheimer disease (AD). The association of plasma amylin with AD in humans is unknown.

**Objectives:**

To measure amylin concentration in plasma by using enzyme-linked immunosorbent assay and to study the association between plasma amylin, incidence of AD, and brain structure in humans.

**Design, Setting, and Participants:**

This cohort study used data from the Framingham Heart Study offspring cohort from 1998 to 2015. Using a Monte Carlo approach, participants were divided into 3 plasma amylin concentration groups: (1) low (<75 pmol/L), (2) high (75-2800 pmol/L), and (3) extremely high (≥2800 pmol/L). Data analyses were conducted October 5, 2017, to December 18, 2018.

**Exposures:**

Baseline plasma amylin concentrations at examination 7.

**Main Outcomes and Measures:**

Incidence of dementia or AD and brain volumetric measures from structural magnetic resonance imaging data.

**Results:**

From the Framingham Heart Study offspring cohort, 3061 participants (mean [SD] age at baseline, 61.0 [9.5] years; 1653 [54.0%] women) who had plasma amylin measurements, dementia incidence, and brain volume measurements on record were included in this study. The distribution of plasma amylin concentrations was highly skewed (median [interquartile range], 7.5 [4.6-18.9] pmol/L; mean [SD], 302.3 [1941.0] pmol/L; range, 0.03-44 623.7 pmol/L). Compared with the low plasma amylin concentration group, the high plasma amylin concentration group had a lower rate of AD incidence (2.3% vs 5.6%; *P* = .04), but the extremely high plasma amylin concentration group had a higher rate of AD incidence (14.3%; *P* < .001). After adjusting for age, sex, education, body mass index, diabetes, cardiovascular disease, high-density lipoprotein level, and *APOE4*, high plasma amylin was not associated with decreased AD risk (hazard ratio, 0.42 [95% CI, 0.16-1.14]; *P* = .09) but was positively associated with volume of gray matter in the temporal lobe (β = 0.17 [SE, 0.05]; *P* < .001). In contrast, extremely high plasma amylin concentration was associated with a higher AD risk (hazard ratio, 2.51 [95% CI, 1.38-4.57]; *P* = .003) but not associated with temporal lobe volume (β = 0.02 [SE, 0.07]; *P* = .82).

**Conclusions and Relevance:**

This study found that plasma amylin concentration was associated with AD incidence and brain structure with a U-shaped pattern. These findings are consistent with preclinical findings that suggest amylin is a neuropeptide that is physiological; however, at extremely high concentrations, it may lead to amylin aggregation and therefore may be a risk factor for AD.

## Key Points

**Question:**

Is plasma amylin concentration associated with Alzheimer disease (AD) risk in humans?

**Findings:**

In this cohort study of 3061 participants in the Framingham Heart Study, the association between plasma amylin concentrations with AD onset and the brain volume was nonlinear and U-shaped. Compared with participants with a low concentration of plasma amylin, participants with a high concentration of plasma amylin had a lower rate of AD incidence but participants with an extremely high concentration of plasma amylin had a higher rate of AD incidence, and a high, but not extremely high, concentration of plasma amylin was positively associated with temporal lobe volumes.

**Meaning:**

Amylin is a neuropeptide that may be beneficial for the aging brain, but an extremely high concentration of plasma amylin, which may lead to amylin aggregation and loss of its protective function for the brain, may be a risk factor for AD.

## Teaser

This cohort study uses data from second-generation participants in the Framingham Heart Study to analyze the association of plasma amylin concentration with Alzheimer disease risk and brain structure.

## Introduction

Amylin, also known as *islet amyloid polypeptide* or *IAPP*, is a highly conserved 37–amino acid peptide produced and secreted with insulin by the β-cells in the pancreas, and it functions as a gut-brain axis hormone.^1,2,3^ Amylin binds to the amylin receptor in the brain^4,5,6,7^ and mediates several important brain functions, including regulating glucose metabolism, modulating inflammatory reactions, and perhaps enhancing neurogenesis.^8,9,10,11,12^ However, amylin can aggregate when concentrations are high and become neurotoxic in cell cultures.^13,14^ A 2017 genome-wide interaction study^15^ showed that an amylin gene polymorphism is associated with brain amyloid burden and cognitive impairment in AD. Amylin aggregations are found colocalized with amyloids in the AD brain.^16^

Amyloid-β peptide (Aβ), a major pathological factor of the AD brain,^17^ and amylin share several features, including binding to the amylin receptor.^18^ Recent preclinical studies^19,20,21^ demonstrated that peripheral treatment of amylin or its analog reduces amyloid burden, tauopathy, and neuroinflammation in the AD brain. It is possible that in the presence of Aβ and when the plasma amylin concentration is low, amylin cannot bind to the amylin receptor sufficiently to effect a protective function for AD^21^; however, when the plasma amylin concentration is extremely high, amylin aggregates and loses its ability to bind to the amylin receptor, in addition to being neurotoxic in the aging brain.^22^ As the longitudinal association of plasma amylin with AD risk and brain structure in humans is unknown, to our knowledge, we hypothesized that the association of plasma amylin concentration with AD risk in humans would be nonlinear.

We used data collected from the Framingham Heart Study (FHS), a large population-based, multigeneration cohort with long and intensive follow-up that includes data on AD development and structural brain volumes.^23^ Included in this study were the FHS offspring cohort (also called *Generation 2* or *Gen 2*) and the plasma samples from examination 7 to measure amylin concentrations. We examined the association of plasma amylin with dementia or AD risk in the following years and with brain volumes, stratified by plasma amylin concentrations.

## Methods

### Study Design and Participants

The FHS is a single-site, community-based, prospective cohort study in Framingham, Massachusetts. The design and selection criteria of the FHS offspring cohort have been previously described.^24^ The baseline for this study was the seventh health examination, which took place from 1998 to 2001, when the enrolled participants underwent cognitive evaluation, medical examination, and blood testing. Follow-up data were collected until 2015.

Participants who did not have dementia, had baseline measurements of plasma amylin, and consented to use of their genetic information (ie, *APOE* genotype) were included in this study. Participants with both *APOE2* and *APOE4* genotypes or who had prevalent dementia at baseline were excluded. We examined the distribution of plasma amylin concentration and its associations with other factors, including demographic characteristics, *APOE* genotype, blood pressure, glucose, and lipid profile. The data on incident dementia, including AD development from examination 7 until 2015, were used to study the association of plasma amylin concentration with AD risk. The data on cardiovascular disease (CVD) and diabetes, in addition to age, sex, and education at examination 7, were used as covariates. Data on a subset of participants who also underwent a brain magnetic resonance imaging (MRI) scan after the seventh examination were also used. Written informed consent was obtained from all study participants, and the study protocol was approved by the Institutional Review Board of Boston University Medical Campus. This study is reported following the Strengthening the Reporting of Observational Studies in Epidemiology (STROBE) reporting guideline. Data analyses were conducted October 5, 2017, to December 18, 2018.

### Plasma Amylin Measurement

Blood testing was conducted after overnight fasting. Blood samples were centrifuged immediately to isolate plasma. We used an enzyme-linked immunosorbent assay (Human Amylin ELISA; EMD Millipore) to measure amylin concentration in plasma according to the manufacturer’s instructions. All samples were assayed in duplicate, and mean final values were generated.

### Dementia or AD Diagnoses

Beginning in 1979, all FHS offspring cohort participants have been followed for incident dementia. Consensus diagnostic procedures have been previously described.^25^ Incidence of dementia, AD, and person-time accruement after the baseline plasma amylin measurement until 2015 were used for analyses.

### Brain Measurements

Brain MRI was conducted beginning in March 1999, and only data that were acquired from MRI after the measurement of plasma amylin concentrations were used. The brain MRI protocol has been reported in detail elsewhere.^26,27^

The semiautomated segmentation protocol for quantifying total cranial volume, total cerebral brain volume, frontal lobar brain volume, parietal lobe brain volume, temporal lobe brain volume, and hippocampal volume has been described elsewhere,^28^ as has the interrater reliabilities for these methods. For segmentation of white matter hyperintensities from other brain tissues, the first and second echo images from T2 sequences were summed and a log-normal distribution was fitted to the summed data. A segmentation threshold for white matter hyperintensities was determined as 1 SD in pixel intensity greater than the mean of the fitted distribution of brain parenchyma. The units for the brain volumes including total cerebral brain volume, frontal lobar brain volume, temporal lobe brain volume, hippocampal volume, and white matter hyperintensities were computed as the percentage of total cranial volume. Each image set underwent rigorous quality control assessment that included assessment of the original acquisition quality as well as the quality of the image processing. Moreover, each of the analysts was highly trained to maintain rigorous precision with intraclass (analyst) coefficients above 90% for all analyses.

### Statistical Analysis

Analyses were performed using SAS statistical software version 9.3 (SAS Institute) and the R statistical environment (R Project for Statistical Computing). Because our preclinical study and others suggested that peripheral amylin treatment is beneficial in mediating the development of AD in the brain,^20,21^ but an extremely high concentration of amylin in amylin transgenic rats has been shown to increase the degree AD development,^29^ we hypothesized that plasma amylin concentration would be associated with AD incidence in a nonlinear pattern. To explore if there was a nonlinear association, we conducted analysis of orthogonal polynomial contrasts and found that tended X linear (log), X^2^ quadratic, X^3^ cubic, and X^4^ quartic effects of plasma amylin concentration on AD incidence (eTable 1 in the Supplement). We then conducted exploratory log-rank analyses with different cutoffs higher than 100 pmol/L in the skewed portion of plasma amylin concentration (eFigure 1 in the Supplement).

To address a nonlinear association with a probable U-shape of plasma amylin concentration with AD, we needed at least 2 cutoff values. However, to our knowledge, there are no references to optimize the cutoff values of plasma amylin to date. Given that plasma amylin concentration was a continuous variable, there might be infinite combinations of choosing the 2 cutoff values if the sample size is very large. We applied Monte Carlo methods, which are a broad class of computational algorithms that rely on repeated random sampling to obtain numerical results. In this study, we split the data set into 2 parts: a training data set and a test data set. We first used the Monte Carlo method to randomly draw a set of cutoff values independently (n = 100 000)^30^ and fit a set of Cox regression models (n = 100 000) based on those randomly chosen cutoff values in the training data set and verified the performance of each model by comparing their area under the curve. We then selected the optimized cutoff values as the ones with maximum area under the curve among all the sets of randomly chosen cutoffs. We estimated 2 optimal cutoff thresholds to generate 3 groups of plasma amylin concentration: (1) low, less than 75 pmol/L; (2) high, 75 to 2800 pmol/L; and (3) extremely high, 2800 pmol/L or more, which were determined by using area under the curve values of an AD incidence prediction model (Cox proportional hazards model) after adjusting for age, sex, and education. We performed 3 analyses for different outcomes. The first analysis was for plasma amylin concentration distribution, demographic characteristics, and medical conditions, in which means and SDs were determined and analysis of variance tests were conducted to compare plasma amylin concentration groups on continuous variables. χ^2^ tests were used to compare plasma amylin concentration groups for categorical variables. Two-tailed *P* values less than .05 were used for statistical significance.

In our second analysis to examine the association of plasma amylin concentration with AD risk, we first used χ^2^ tests to examine AD incidence. Then the log-rank test was applied to compare the onset of AD based on the 3 plasma amylin concentration groups. Cox proportional hazards models were used to evaluate AD risk after adjusting for confounders. Two-tailed *P* values less than .05 were used for statistical significance.

In our third analysis to examine the association of plasma amylin concentration with brain volumes, we used 2148 participants who had brain MRI data from around or after the date of plasma amylin measurement for the brain volume comparisons. We used the mean brain volumes, including gray matter and white matter, measured in centimeters cubed, divided by total cerebral brain volume to study brain atrophy. Using analysis of variance, the association of plasma amylin concentration with brain volume was examined. Next, multivariate linear regression was used to study the association of different plasma amylin concentration with regional brain volume measures as an outcome controlling for confounders. Given multiple testing and the need to minimize the rate of false positives, Bonferroni-corrected 2-tailed *P* values were used with statistical significance set at less than .004.

While the skewed portion of plasma amylin concentration might have been associated with AD incidence, the normal distributed portion of plasma amylin concentration might have been associated with physiological factors because amylin is a hormone. For additional analysis, we divided the low plasma amylin concentration group into quintiles. Quintiles (Q) were defined as Q1, less than 4.2 pmol/L; Q2, 4.2 to 5.9 mol/L; Q3, 6.0 to 9.4 pmol/L; Q4, 9.5 to 26.9 pmol/L; and Q5, more than 27.0 pmol/L.

## Results

### Characterization of Plasma Amylin Concentration Among FHS Participants

The FHS offspring cohort at examination 7 enrolled 3539 study participants; 3226 participants (91.2%) underwent blood testing and had plasma amylin measurements; and of them, 3137 participants (97.2%) consented to have their *APOE* genetic information examined. After excluding 60 individuals with both *APOE2* and *APOE4* and 16 individuals who had prevalent dementia at baseline, 3061 participants (mean [SD] age, 61.0 [9.5] years; 1653 [54.0%] women) were included in this study (Table 1). The distribution of plasma amylin concentrations was highly skewed toward to the high extreme, with a median (interquartile range) of 7.5 (4.6-18.9) pmol/L, a mean (SD) of 302.3 (1941.0) pmol/L, and a total range of 0.03 pmol/L to 44 623.7 pmol/L. After logarithmizing the plasma amylin data and conducting distribution plots, there were 2752 participants (89.9%) in the low plasma amylin concentration group, 222 participants (7.3%) in the high plasma amylin concentration group, and 87 participants (2.8%) in the extremely high plasma amylin concentration group (eFigure 2 in the Supplement). There was no difference between men and women with respect to the distribution of plasma amylin concentration.

**Table 1.** Characteristics of the Study Sample Based on Plasma Amylin Concentration

Characteristic	Mean (SD)				*df*	*F* Score	*P* Value
	Total (N = 3061)	Low Plasma Amylin (n = 2752)^a^	High Plasma Amylin (n = 222)^b^	Extremely High Plasma Amylin (n = 87)^c^			
Age, y	61.0 (9.5)	61.1 (9.5)	59.8 (9.0)	61.8 (10.4)	2	2.34	.10
Education, y	14.1 (2.6)	14.1 (2.6)	14.4 (2.7)	14.0 (2.6)	2	1.58	.21
Women, No. (%)	1653 (54.0)	1494 (54.3)	111 (50.0)	48 (55.2)	2	1.57	.46
BMI	28.1 (5.3)	28.2 (5.4)	27.8 (5.1)	27.0 (4.2)	2	2.50	.08
Waist circumference, cm	99.8 (14.0)	99.8 (14.0)	99.1 (14.0)	97.0 (11.9)	2	1.94	.14
Hip circumference, cm	105.2 (10.4)	105.2 (10.7)	105.2 (9.7)	102.9 (9.1)	2	2.16	.12
SBP, mm Hg	126.9 (18.7)	127.0 (18.8)	126.0 (18.8)	127.2 (18.4)	2	0.26	.77
DBP, mm Hg	74.0 (9.7)	74.0 (9.8)	73.7 (9.6)	74.2 (9.0)	2	0.13	.88
Diabetes, No. (%)	343 (11.2)	312 (11.3)	21 (9.5)	10 (11.5)	2	0.74	.69
CVD, No. (%)	300 (11.3)	273 (11.5)	21 (10.7)	6 (7.4)	2	1.40	.50
Glucose, mg/dL	104.2 (26.4)	104.2 (26.3)	103.1 (26.8)	105.0 (30.1)	2	0.22	.80
Total cholesterol, mg/dL	200.5 (36.8)	200.4 (36.7)	202.0 (37.8)	198.2 (36.2)	2	0.36	.70
Triglycerides, mg/dL	137.0 (88.7)	136.8 (88.5)	139.8 (99.3)	134.7 (63.3)	2	0.15	.86
LDL, mg/dL	119.6 (32.7)	119.6 (32.7)	122.5 (33.8)	120.7 (31.2)	2	0.79	.45
HDL, mg/dL	53.9 (17.0)	54.1 (17.0)	52.6 (17.4)	50.4 (15.4)	2	2.64	.07
Creatinine, mg/dL	0.9 (0.3)	0.9 (0.3)	0.9 (0.2)	0.8 (0.2)	2	0.98	.37
CRP, mg/L	4.3 (7.6)	4.2 (6.2)	5.3 (17.9)	3.4 (4.3)	2	2.66	.07
*APOE4*, No. (%)	634 (20.7)	570 (20.7)	41 (18.5)	23 (26.4)	2	2.42	.30

Abbreviations: BMI, body mass index (calculated as weight in kilograms divided by height in meters squared); CRP, C-reactive protein; CVD, cardiovascular disease; DBP, diastolic blood pressure; HDL, high-density lipoprotein; LDL, low-density lipoprotein; SBP, systolic blood pressure.

SI conversion factors: To convert glucose to millimoles per liter, multiply by 0.0555; total cholesterol, LDL, and HDL to millimoles per liter, multiply by 0.0259; triglycerides to millimoles per liter, multiply by 0.0113; creatinine to micromoles per liter, multiply by 88.4; and CRP to nanomoles per liter, multiply by 9.524.

^a^ Plasma amylin concentration, less than 75 pmol/L.

^b^ Plasma amylin concentration, 75 pmol/L to less than 2800 pmol/L.

^c^ Plasma amylin concentration, 2800 pmol/L or greater.

### Associations of Plasma Amylin Concentrations With Alzheimer Disease Risk

The mean (SD) follow-up for participants after the measurement of plasma amylin was 14.8 (3.8) years. During this time, 210 participants (6.9%) developed dementia and 169 participants (6.6%) had AD. Among the 3 plasma amylin concentration groups, compared with the low plasma amylin concentration group, the high plasma amylin concentration group had lower risks of dementia (6.7% vs 4.5%) and AD (5.6% vs 2.3%), but the extremely high plasma amylin concentration group had higher risks of dementia (17.2%) and AD (14.3%) (Table 2). Using a log-rank test and the low plasma amylin concentration group as the reference, we found that participants with a high concentration of plasma amylin had a lower risk for the onset of AD (*P* = .04), but participants with an extremely high concentration of plasma amylin had a higher risk of the onset of AD (*P* = .002) (Figure).

**Table 2.** Comparisons of AD Incidence Stratified by Plasma Amylin Concentration

Subgroup	No. (%)			*df*	Log-Rank Test	*P* Value
	Low Plasma Amylin (n = 2752)^a^	High Plasma Amylin (n = 222)^b^	Extremely High Plasma Amylin (n = 87)^c^			
Dementia	185 (6.7)	10 (4.5)	15 (17.2)	2	15.4	<.001
AD incidence	152 (5.5)	5 (2.3)	12 (13.8)	2	15.7	<.001
Men	1258 (45.7)	111 (50.0)	39 (44.8)			
AD incidence	59 (4.7)	1 (0.9)	2 (5.1)	2	3.80	.20
Women	1494 (54.3)	111 (50.0)	48 (55.2)			
AD incidence	93 (6.2)	4 (3.6)	10 (20.8)	2	17.7	<.001
No diabetes	2437 (88.7)	201 (90.5)	77 (88.5)			
AD incidence	117 (4.8)	4 (2.0)	9 (11.7)	2	11.3	.004
Diabetes	312 (11.3)	21 (9.5)	10 (11.5)			
AD incidence	35 (11.2)^d^	1 (4.8)	3 (30.0)	2	4.1	.10
*APOE2*	380 (13.8)	24 (10.8)	9 (10.3)			
AD incidence	14 (3.7)	1 (4.2)	1 (11.1)	2	1.10	.60
*APOE3*	1802 (65.5)	157 (70.7)	55 (63.2)			
AD incidence	83 (4.6)	3 (1.9)	6 (10.9)	2	7.60	.02
*APOE4*	570 (20.7)	41 (18.5)	23 (26.4)			
AD incidence	55 (9.6)^d^	1 (2.4)	5 (21.7)	2	6.20	.05

Abbreviation: AD, Alzheimer disease.

^a^ Plasma amylin concentration, less than 75 pmol/L.

^b^ Plasma amylin concentration, 75 pmol/L to less than 2800 pmol/L.

^c^ Plasma amylin concentration, 2800 pmol/L or greater.

^d^ *P* < .001 for the column comparisons of AD incidence among plasma amylin concentration groups.

**Figure.**
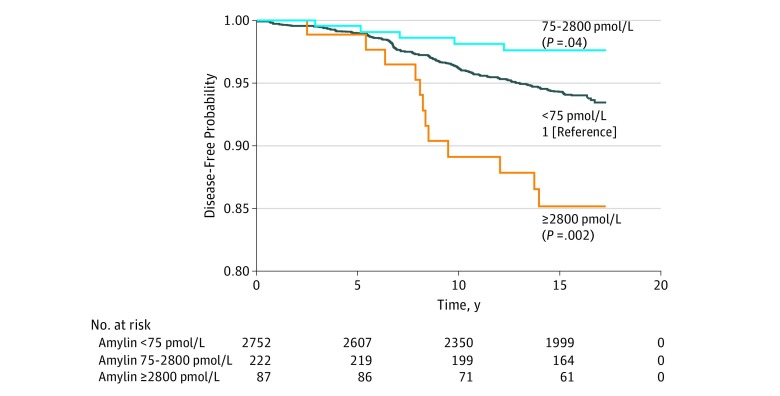
Log-Rank Analysis for Alzheimer Disease–Free or Dementia-Free Survival Among 3 Groups of Plasma Amylin Concentration

After adjusting for age; sex; education; body mass index (BMI), calculated as weight in kilograms divided by height in meters squared; diabetes; CVD; *APOE4* genotype; and high-density lipoprotein, we found that extremely high plasma amylin concentration was associated with 2- to 3-fold increased AD incidence during the follow-up compared with low plasma amylin concentration (hazard ratio, 2.51 [95% CI, 1.38-4.57]; *P* = .003) (Table 3). The association of high plasma amylin concentration with AD incidence did not reach statistical significance (hazard ratio, 0.42 [95% CI, 0.16-1.14]; *P* = .09) (Table 3). Participants were further stratified into men and women; those without or with diabetes; and those with *APOE2*, *APOE3*, or *APOE4* genotypes. Similar U-shaped associations of the plasma amylin cutoffs with AD risk were found in these analyses, except for *APOE2* (Table 2).

**Table 3.** Association of Plasma Amylin Concentration With Risk of Alzheimer Disease in Multivariate Cox Regression Model

Plasma Amylin Concentration	Hazard Ratio (95% CI)^a^	*P* Value
High^b^	0.42 (0.16-1.14)	.09
Extremely high^c^	2.51 (1.38-4.57)	.003

^a^ Low plasma amylin concentration (<75 pmol/L) was used as a reference level. Cox regression was adjusted for confounders, including age, sex, education, *APOE4*, body mass index, diabetes, cardiovascular disease, and high-density lipoprotein.

^b^ 75 pmol/L to less than 2800 pmol/L.

^c^ 2800 pmol/L or greater.

Other variables among the 3 amylin groups did not show statistical significance (Table 1). In contrast, across the quintiles of plasma amylin, there were statistically significant differences of mean (SD) BMI (Q1, 27.6 [5.0]; Q2, 28.2 [5.2]; Q3, 28.7 [5.8]; Q4, 28.4 [5.6]; Q5, 27.7 [4.9]; *P* = .001), waist circumference (Q1, 98.6 [13.7] cm; Q2, 99.8 [13.7] cm; Q3, 101.3 [14.7] cm; Q4, 100.3 [14.2] cm; Q5, 98.6 [13.7] cm; *P* = .002), diastolic blood pressure (Q1, 72.9 [9.4] mm Hg; Q2, 74.1 [9.7] mm Hg; Q3, 74.4 [9.6] mm Hg; Q4, 74.6 [10.2] mm Hg; Q5, 74.0 [9.6] mm Hg; *P* = .02), triglyceride (Q1, 121.8 [71.9] mg/dL; Q2, 142.3 [78.7] mg/dL; Q3, 144.6 [98.1] mg/dL; Q4, 138.5 [93.6] mg/dL; Q5, 137.7 [96.7] mg/dL; *P* < .001), and high-density lipoprotein (Q1, 56.8 [16.6] mg/dL; Q2, 53.0 [16.0] mg/dL; Q3, 52.9 [17.2] mg/dL; Q4, 53.6 [17.6] mg/dL; Q5, 53.1 [17.2] mg/dL; *P* < .001) (eTable 2 in the Supplement). However, the associations among amylin quintiles and these variables were nonlinear with a slight U or inverted U shape, eg, the plasma amylin concentration quintile with the lowest or largest mean was not the lowest or the highest plasma amylin concentration. We did not find statistically significant differences in AD incidence among the quintiles (eTable 3 in the Supplement), possibly owing to the U-shaped association of plasma amylin concentration with AD risk.

### Associations of Plasma Amylin and Brain Volumes

The study participants who had undergone brain MRI were younger compared with those who had not (mean [SD] age, 60.6 [9.4] years vs 62.1 [9.6] years; *P* < .001) and had no statistically significant difference in sex (1151 women [54.0%] of 2148 participants with brain MRI data vs 502 women [47.9%] of 913 participants without brain MRI data; *P* = .50). There was no statistically significant difference in cumulative AD incidence between participants who had or had not undergone brain MRI (124 of 2148 participants [5.8%] with brain MRI data vs 45 of 913 participants [5.0%] without brain MRI data; *P* = .40). Using the Monte Carlo approach for cutoffs of plasma amylin concentration, we also found an inverted U– or U-shaped association of plasma amylin concentration with brain volume. Compared with participants with low plasma amylin concentration, participants with high plasma amylin concentration had higher mean (SD) volumes as percentages of total cerebral brain volume for temporal lobe brain volume (13.8% [0.7%] vs 13.6% [0.8%]) and temporal lobe gray matter volume (8.5% [0.5%] vs 8.4% [0.5%]) and lower median (interquartile range) volume of white matter hyperintensities (0.8% [0.4%-1.6%] vs 1.1% [0.5%-2.2%]), but participants with an extremely high plasma amylin concentration had similar volumes of these brain regions (eTable 4 in the Supplement). Using multivariate linear regression analyses after adjusting for age, sex, education, BMI, diabetes, CVD, and *APOE4*, the high concentration of plasma amylin cutoff remained positively associated with temporal lobe brain gray matter volume (β = 0.17 [SE, 0.05]; *P* < .001) (Table 4). In the same models, extremely high plasma amylin concentration was not found to be associated with temporal lobe brain volume or white matter hyperintensities.

**Table 4.** General Linear Regression Analyses of the Association of Plasma Amylin Concentration With Brain Volume^a^

Plasma Amylin Concentration	Temporal Lobe Brain Gray Matter Volume, %^b^		Logarithm of White Matter Hyperintensities, %^b^	
	β Estimate (SE)	*P* Value^c^	β Estimate (SE)	*P* Value^c^
High^d^	0.17 (0.05)	<.001	−1.85 (0.70)	.008
Extremely high^e^	0.02 (0.07)	.82	0.63 (1.09)	.57

^a^ The models were adjusted for age, sex, education, smoking, body mass index, diabetes, cardiovascular diseases, *APOE4*, and high-density lipoprotein. Low plasma amylin concentration (<75 pmol/L) was used as the reference.

^b^ Given as a percentage of total cerebral brain volume.

^c^ *P* values adjusted using conventional Bonferroni correction (*P* < .004).

^d^ 75 pmol/L to less than 2800 pmol/L.

^e^ 2800 pmol/L or greater.

The brain volumes among plasma amylin concentration quintiles were also compared (eTable 5 in the Supplement). Using analysis of variance, we found that the median (interquartile range) volume of white matter hyperintensities was negatively associated with increasing quintiles of plasma amylin (Q1, 1.1 [0.5-2.3]; Q2, 1.2 [0.5-2.4]; Q3, 0.93 [0.4-2.0]; Q4, 1.1 [0.5-2.1]; Q5, 1.0 [0.5-2.1]; *P* = .007). Using multivariate linear regression analyses after adjusting for age, sex, education, BMI, diabetes, CVD, and *APOE4*, the largest amylin quintile, Q5, remained positively associated with temporal lobe brain gray matter volume (β = 0.10 [SE, 0.04]; *P* = .007) (eTable 6 in the Supplement). In the same model, we found that Q5 was negatively associated with the logarithm of white matter hyperintensities (β = −1.12 [SE, 0.57]; *P* = .05). However, these were not statistically significant after Bonferroni correction.

## Discussion

The U-shaped association of plasma amylin concentration with AD incidence was consistent with previous preclinical findings. Compared with low plasma amylin concentration, participants with high plasma amylin concentration had a lower rate of AD incidence. Consistently, high plasma amylin concentration had a protective association for temporal lobe brain volume. Although the mechanism of amylin’s beneficial effects for the brain is still unclear, amylin is a gut-brain axis hormone that is produced by β-cells in the pancreas^31^ and crosses the blood-brain barrier.^4,5,6^ The beneficial effects of amylin for AD are likely through binding to the amylin receptor,^21^ which is one type of G-protein coupled receptor and is composed of the calcitonin receptor combined with different receptor activity–modifying proteins.^7,32^

In contrast to the high range of plasma amylin, the extremely high range of plasma amylin concentration was associated with increased AD risk in long-term follow-up and did not show any association with temporal lobe brain volume. There are 2 possible mechanisms for this: (1) amylin aggregates and causes neurotoxic effects, or (2) aggregated amylin cannot bind to its cognate amylin receptor to mediate the protective function against AD.^33^ Our recent in vitro study showed that exogenously adding human amylin was beneficial to antagonize the neurotoxic effects caused by aggregated Aβ1-42, but adding an extremely high concentration of human amylin was ineffective and synergetic with Aβ’s neurotoxicity in primary rat cortical neurons.^34^ Meanwhile, a 2017 study^35^ demonstrated that low amylin concentrations vs high amylin concentrations bind to different receptors, and we contend that one is neurophysiological and the other neurotoxic, each through independent pathways. At an extremely high plasma amylin concentration, probably in nanomole-per-liter ranges, for a long duration, there may be an independent pathway for AD pathogenesis stemming from amylin’s physiological effects as a hormone. The extremely high plasma amylin concentrations were associated with increased AD incidence during the study follow-up. Since case-control studies have shown that patients with AD have a lower concentration of plasma amylin than controls^19,33^ and amylin aggregates are found in the AD brain,^16^ it is possible that an extremely high plasma amylin concentration declines owing to its aggregation and deposition in the brain during neurodegeneration processed in the brain occurring for longer than 10 years.

It has been overwhelmingly illustrated in multiple cohort studies with large and small sample sizes^36^ that *APOE4* is a major genetic risk factor, while *APOE2* is a protecting factor for AD. In our study, when plasma amylin concentration was high and beneficial, *APOE4* was not a risk factor for AD; when plasma amylin concentration was extremely high and was associated with increased risk of AD, *APOE2* was not associated with protection from AD. A 2008 in vitro experiment^37^ found that *APOE4* could bind amylin, which suggests that amylin may be an important player interacting with the *APOE* genotype in the pathogenesis of late-onset AD. In contrast, being a woman was a risk factor for AD across the 3 amylin groups. It has been widely demonstrated that rat amylin, with 3 amino acid differences from human amylin, does not have aggregating characteristics.^13^ Pramlintide, an amylin analog based on rat amylin sequence so that it cannot aggregate, is a US Food and Drug Administration–approved and effective drug for type 2 diabetes^38^ with a favorable safety profile in clinical use.^39^ Thus, pramlintide could be investigated as a repurposed drug for AD for patients who have a low plasma amylin concentration.

### Limitations

This study has limitations. Since participants were relatively young and only a small number had dementia when plasma amylin concentrations were measured, we did not have enough power to study the prevalent association of plasma amylin with AD for this study. We also did not have longitudinal measurements of plasma amylin; thus, we were unable to determine whether plasma amylin concentration changes during AD pathogenesis. Another limitation is that this study sample did not include people of different ethnicities. This U-shaped association between plasma amylin and long-term risk of AD needs to be examined in other human study populations.

## Conclusions

The distribution of plasma amylin concentration was skewed in humans and associated with AD and brain structures in a U-shaped manner. It is important to monitor plasma amylin concentration for elderly patients, especially *APOE4* carriers, women, and people with diabetes, for the sequela of AD development. Future studies are needed to determine whether the similar associations of plasma amylin with AD that have been found in other cohort studies and whether amylin analogs can be repurposed as a therapeutic for AD, especially people with *APOE4*, women, and people with diabetes.

## Notes

Supplement. **eTable 1.** Analysis of Orthogonal Polynomial Contrasts and Cox Proportional Hazard Regression on the Association of Plasma Amylin Concentration With Risk of Alzheimer Disease **eTable 2.** General Characterization of the Study Sample Based on Plasma Amylin Concentration Quintiles **eTable 3.** Association of Plasma Amylin Concentration Quintiles With Alzheimer Disease **eTable 4.** Brain Volume Comparisons Among Patients With a Low, High, or Extremely High Plasma Amylin Concentration **eTable 5.** Characterization of Brain Volume Based on Plasma Amylin Concentration Quintiles **eTable 6.** General Linear Regression Analyses on the Association of Plasma Amylin Concentration With Brain Volume **eFigure 1.** Accumulative Alzheimer Disease Incidence Rates Based on Different Cutoffs for Plasma Amylin Concentration **eFigure 2.** Distribution of Plasma Amylin Concentrations Among the Population of the Framingham Heart Study
