# Supplementary material for: Association of Plasma Amylin Concentration With Alzheimer Disease and Brain Structure in Older Adults
Source: JAMA Netw Open. 2019 Aug 21;2(8):e199826. doi: 10.1001/jamanetworkopen.2019.9826 (PMC6707010; doi:10.1001/jamanetworkopen.2019.9826)
Supplement: Supplement. — eTable 1. Analysis of Orthogonal Polynomial Contrasts and Cox Proportional Hazard Regression on the Association of Plasma Amylin Concentration With Risk of Alzheimer Disease eTable 2. General Characterization of the Study Sample Based on Plasma Amylin Concentration Quintiles eTable 3. Association of Plasma Amylin Concentration Quintiles With Alzheimer Disease eTable 4. Brain Volume Comparisons Among Patients With a Low, High, or Extremely High Plasma Amylin Concentration eTable 5. Characterization of Brain Volume Based on Plasma Amylin Concentration Quintiles eTable 6. General Linear Regression Analyses on the Association of Plasma Amylin Concentration With Brain Volume eFigure 1. Accumulative Alzheimer Disease Incidence Rates Based on Different Cutoffs for Plasma Amylin Concentration eFigure 2. Distribution of Plasma Amylin Concentrations Among the Population of the Framingham Heart Study [file jamanetwopen-2-e199826-s001.pdf]

Supplementary Online Content

Zhu H, Tao Q, Ang TFA, et al. Association of plasma amylin concentration with Alzheimer disease and brain structure in older adults. *JAMA Netw Open*. 2019;2(8):e199826. doi:10.1001/jamanetworkopen.2019.9826

eTable 1. Analysis of Orthogonal Polynomial Contrasts and Cox Proportional Hazard Regression on the Association of Plasma Amylin Concentration With Risk of Alzheimer Disease
eTable 2. General Characterization of the Study Sample Based on Plasma Amylin Concentration Quintiles
eTable 3. Association of Plasma Amylin Concentration Quintiles With Alzheimer Disease
eTable 4. Brain Volume Comparisons Among Patients With a Low, High, or Extremely High Plasma Amylin Concentration
eTable 5. Characterization of Brain Volume Based on Plasma Amylin Concentration Quintiles
eTable 6. General Linear Regression Analyses on the Association of Plasma Amylin Concentration With Brain Volume
eFigure 1. Accumulative Alzheimer Disease Incidence Rates Based on Different Cutoffs for Plasma Amylin Concentration
eFigure 2. Distribution of Plasma Amylin Concentrations Among the Population of the Framingham Heart Study

This supplementary material has been provided by the authors to give readers additional information about their work.

© 2019 Zhu H et al. *JAMA Network Open.*

eTable 1. Analysis of Orthogonal Polynomial Contrasts and Cox Proportional Hazard Regression on the Association of Plasma Amylin Concentration With Risk of Alzheimer Disease

**Multivariate COX regression**	**Hazard Ratio (95% CI)**	**P values**
**All participants (n =3020)**		
X	64.14 (0.36, >100)	0.12
X^2	0.04 (<0.01, 3.58)	0.16
X^3	2.58 (0.59, 11.3)	0.21
X^4	0.91 (0.78, 1.07)	0.27
**Male (n =1384)**		
X	>100 (4.25, >100)	0.02
X^2	< 0.01 (<0.01, 0.31)	0.03
X^3	28.51 (1.29, >100)	0.03
X^4	0.70 (0.50, 1.00)	0.05
**Female (n =1636)**		
X	2.50 (0.01, >100)	0.76
X^2	0.68 (<0.01, >100)	0.88
X^3	1.07 (0.21, 5.50)	0.93
X^4	1.00 (0.84, 1.19)	0.99

The analysis of orthogonal polynomial contrasts and cox model (Proportional hazards regression) were conducted to explore a nonlinear association between plasma amylin and AD risk in all participants (n = 3020), and in males (n = 1384) vs. in females (n = 1636). Four single degree of freedom orthogonal polynomial contrasts of plasma amylin were generated: the log (base 10) transform of plasma amylin: X = log10 (amylin); the quadratic transform of log10 (amylin): X^2, the cubic of log10 (amylin): X^3, and the quartic of log10 (amylin): X^4 and used in cox models on the incidence of new AD cases after adjustment for confounders including age, sex, and eduction.

© 2019 Zhu H et al. *JAMA Network Open.*

eTable 2. General Characterization of the Study Sample Based on Plasma Amylin Concentration Quintiles

Demographics and medical conditions	Quintile 1 < 4.2 n=609	Quintile 2 4.2-5.9 n=616	Quintile 3 6.0-9.4 n=617	Quintile 4 9.5-27.0 n=607	Quintile 5 ≥ 27.0 n=612	Df	F	P
**Age7 (years), mean ± SD**	61.4 ± 9.4	61.6 ± 9.3	60.2 ± 9.8	61.1 ± 9.5	60.8 ± 9.4	4	2.05	0.09
**Education (years), mean ± SD**	13.9 ± 2.5	14.1 ± 2.5	14.0 ± 2.7	14.2 ± 2.7	14.4 ± 2.6	4	2.31	0.06
**Female, n (% of total n)**	342 (55.8)	332 (54.2)	318 (51.9)	343 (56.1)	318 (52)	4	4.07	0.40
**BMI (kg/m^2), mean ± SD**	27.6 ± 5	28.2 ± 5.2	28.7 ± 5.8	28.4 ± 5.6	27.7 ± 4.9	4	4.99	0.001
**WAIST (inch), mean ± SD**	38.8 ± 5.4	39.3 ± 5.4	39.9 ± 5.8	39.5 ± 5.6	38.8 ± 5.4	4	4.31	0.002
**HIP (inch), mean ± SD**	41.1 ± 4.0	41.5 ± 4.3	41.7 ± 4.4	41.5 ± 4.0	41.2 ± 3.9	4	2.01	0.09
**SBP (mmHg), mean ± SD**	126.0 ± 18.6	127.8 ± 18	126.2 ± 19.8	128.1 ± 19.2	126.5 ± 18.1	4	1.57	0.18
**DBP (mmHg), mean ± SD**	72.9 ± 9.4	74.1 ± 9.7	74.4 ± 9.6	74.6 ± 10.2	74.0 ± 9.6	4	2.88	0.02
**Diabetes, n (% of total n)**	71 (11.6)	67 (10.9)	73 (11.9)	70 (11.5)	62 (10.1)	4	1.22	0.88
**CVD, n (% of total n)**	68 (12.9)	63 (11.8)	62 (11.9)	50 (9.5)	57 (10.5)	4	3.64	0.46
**Glucose (mg/dL), mean ± SD**	102.7 ± 23.8	103.9 ± 25.1	105.2 ± 27.6	105.2 ± 27.6	103.8 ± 27.9	4	0.95	0.44
**Total cholesterol (mg/dL), mean ± SD**	198.6 ± 35.8	203.2 ± 37.2	201.4 ± 37.9	199.4 ± 36.6	199.8 ± 36.2	4	1.55	0.19
**Triglyceride (mg/dL), mean ± SD**	121.8 ± 71.9	142.3 ± 78.7	144.6 ± 98.1	138.5 ± 93.6	137.7 ± 96.7	4	6.28	<0.001
**LDL (mg/dL), mean ± SD**	117.6 ± 31.5	122.2±32.9	120.8 ± 35.3	118.7 ± 31.6	120.0 ± 32.2	4	1.80	0.13

© 2019 Zhu H et al. *JAMA Network Open.*

**HDL(mg/dL), mean ± SD**	56.8 ± 16.6	53.0 ± 16.0	52.9 ± 17.2	53.6 ± 17.6	53.1 ± 17.2	4	5.90	<0.001
**Creatinine (mg/dL), mean ± SD**	0.8 ± 0.2	0.9 ± 0.4	0.9 ± 0.4	0.9 ± 0.3	0.9 ± 0.2	4	1.85	0.12
**CRP (mg/dL), mean ± SD**	3.7 ± 4.7	4.7 ± 6.9	4.4 ± 6.8	4.1 ± 5.3	4.5 ± 12	4	1.52	0.20
**ApoE4, n (% of total n)**	125 (20.4)	130 (21.2)	145 (23.7)	113 (18.5)	121 (19.8)	4	5.53	0.24

BMI = Body mass index; SBP = systolic blood pressure; DBP = diastolic blood pressure; CVD = cardiovascular disease; LDL = low density lipoprotein; HDL = high density lipoprotein; CRP = C-reactive protein; ApoE4 = apolipoprotein E4.
3061 subjects were divided based on plasma amylin quintiles. Mean ± SD with one-way ANOVA was used to test differences in the variables among amylin quintiles, median (Q1, Q3) with Kruskal-Wallis test was applied when a concentration distribution was skewed. And Chi-square (χ^2) test was used to compare counts, n/total (%). P values for statistical significance are shown for the comparisons.

© 2019 Zhu H et al. *JAMA Network Open.*

eTable 3. Association of Plasma Amylin Concentration Quintiles With Alzheimer Disease

	Amylin, pM							
**Cognitive incidence**	**Quintile 1<br>< 4.2<br>n = 609**	**Quintile 2<br>4.2-5.9<br>n = 616**	**Quintile 3<br>6.0-9.4<br>n = 617**	**Quintile 4<br>9.5-26.9<br>n = 607**	**Quintile 5<br>≥ 27.0<br>n = 612**	**Df**	**F**	**P**
**Dementia incidence, n (% of total n)**	40 (6.6)	38 (6.2)	42 (6.8)	42 (6.9)	48 (7.8)	4	1.10	0.90
**AD incidence, n (% of total n)**	30 (4.9)	32 (5.2)	33 (5.3)	36 (5.9)	38 (6.2)	4	1.10	0.90

AD = Alzheimer's disease

Participants who had plasma amylin measurements were used to divide into quintiles. Fisher exact test was used for the prevalence comparisons, and log-rank test was used for the incidence comparisons of dementia and AD.

© 2019 Zhu H et al. *JAMA Network Open.*

eTable 4. Brain Volume Comparisons Among Patients With a Low, High, or Extremely High Plasma Amylin Concentration

**Brain volumes**	**Amylin < 75 pM n = 1938**	**Amylin ≥ 75 to < 2800 pM n = 152**	**Amylin ≥ 2800 pM n = 58**	**Df**	**F**	**P**
**Gray/TCBV %, mean±SD**	41.4 ± 1.9	41.7±1.7	41.5±1.9	2	3.30	0.04
**TBV/TCBV %, mean±SD**	13.6±0.8	13.8±0.7	13.6±0.7	2	4.14	0.02
**TBV Gray/TCBV %, mean±SD**	8.4±0.5	8.5±0.5	8.4±0.5	2	5.74	0.003
**HBV/TCBV %, mean±SD**	0.47±0.04	0.47±0.04	0.48±0.05	2	3.29	0.04
**WMHI, median (Q1, Q3)**	1.07 (0.48, 2.19)	0.82 (0.42, 1.56)	1.14 (0.58, 2.50)	2	3.85	0.15

TCBV = total cerebral brain volume; FBV = frontal lobe brain volume; TBV = temporal lobe brain volume; HPV = hippocampal volume; WMHI = white matter hyperintensities volume. 2148 participants who had plasma amylin measurements and a brain MRI were used to divide into quintiles (A) and into three cut-off based groups (B). Mean ± SD with one-way ANOVA was used to test brain volume differences among amylin quintiles, median (Q1, Q3) with Kruskal-Wallis test was applied to WMHI due to its skewed distribution. P values adjusted by using conventional Bonferroni correction ($p < 0.004$) were used.

© 2019 Zhu H et al. *JAMA Network Open.*

eTable 5: Characterization of Brain Volume Based on Plasma Amylin Concentration Quintiles

**Brain volumes, mean ± SD**	**Quintile 1 < 4.2 n = 420**	**Quintile 2 4.2-5.9 n = 440**	**Quintile 3 6.0-9.4 n = 423**	**Quintile 4 9.5-26.9 n = 435**	**Quintile 5 ≥ 27.0 n = 430**	**Df**	**F**	**P**
**TCBV**	1408.8 ± 144.3	1407.6 ± 136.4	1416 ± 140.7	1418.1 ± 140.4	1419 ± 141.7	4	0.62	0.65
**Gray/TCBV %**	41.3 ± 1.8	41.3 ± 1.8	41.4 ± 1.9	41.4 ± 2.0	41.6 ± 1.8	4	1.60	0.17
**White/TCBV %**	34.4 ± 2.5	34.5 ± 2.5	34.6 ± 2.5	34.4 ± 2.5	34.4 ± 2.4	4	0.48	0.75
**FBV/TCBV %**	23.6 ± 1.2	23.6 ± 1.3	23.7 ± 1.3	23.7 ± 1.4	23.6 ± 1.3	4	0.87	0.48
**FBV Gray/TCBV %**	11.9 ± 0.7	11.9 ± 0.7	12.0 ± 0.8	12.0 ± 0.8	12.0 ± 0.7	4	1.28	0.27
**FBV White/TCBV %**	11.7 ± 1.0	11.7 ± 1.0	11.7 ± 1.0	11.7 ± 1.0	11.6 ± 1.0	4	0.90	0.46
**PBV/TCBV %**	13.0 ± 0.7	13.0 ± 0.7	13.0 ± 0.8	13.0 ± 0.7	13.0 ± 0.8	4	0.22	0.93
**PBV Gray/TCBV %**	6.7 ± 0.4	6.7 ± 0.4	6.7 ± 0.5	6.7 ± 0.4	6.7 ± 0.4	4	0.48	0.75
**PBV White/TCBV %**	6.4 ± 0.5	6.3 ± 0.6	6.4 ± 0.6	6.3 ± 0.5	6.3 ± 0.5	4	1.17	0.32
**TBV/TCBV %**	13.5 ± 0.8	13.7 ± 0.8	13.6 ± 0.8	13.6 ± 0.8	13.7 ± 0.8	4	2.39	0.05
**TBV Gray/TCBV %**	8.3 ± 0.5	8.4 ± 0.5	8.4 ± 0.5	8.4 ± 0.5	8.4 ± 0.5	4	2.34	0.05
**TBV White/TCBV %**	5.2 ± 0.5	5.3 ± 0.5	5.3 ± 0.5	5.3 ± 0.5	5.3 ± 0.5	4	1.11	0.34
**HBV/TCBV %**	0.47 ± 0.04	0.47 ± 0.04	0.47 ± 0.04	0.47 ± 0.04	0.47 ± 0.05	4	0.76	0.55
**WMHI**	1.14 (0.54, 2.33)	1.21 (0.53, 2.42)	0.93 (0.43, 1.97)	1.05 (0.47, 2.09)	0.95 (0.47, 2.08)	4	14.02	0.007

TCBV = total cerebral brain volume; FBV = frontal lobe brain volume; TBV = temporal lobe brain volume; HPV = hippocampal volume; WMHI = white matter hyperintensities volume. 2148 participants who had plasma amylin measurements and a brain MRI were used to divide into quintiles (A) and into three cut-off based groups (B). Mean ± SD with one-way ANOVA was used to test brain volume differences among amylin quintiles, median (Q1, Q3) with Kruskal-Wallis test was applied to WMHI due to its skewed distribution. P values adjusted by using conventional Bonferroni correction (p < 0.004) were used.

© 2019 Zhu H et al. *JAMA Network Open.*

eTable 6: General Linear Regression Analyses on the Association of Plasma Amylin Concentration With Brain Volume

Amylin concentrations		TBV gray matter/TCBV% n = 2148			Logarithm of WMHI/TCBV % n = 2148	
		β Estimate ± SE	p values		β Estimate ± SE	p values
**Q1**		Reference	-		Reference	-
**Q2**		0.06 ± 0.04	0.11		0.39 ± 0.57	0.49
**Q3**		0.02 ± 0.04	0.64		-0.53 ± 0.58	0.36
**Q4**		0.04 ± 0.04	0.36		-0.56 ± 0.57	0.33
**Q5**		0.10 ± 0.04	0.007		-1.11 ± 0.57	0.05

TCBV = total cerebral brain volume; TBV = Temporal lobe brain volume; WMHI = white matter hyperintensities.
General linear regression (GLM) was used to study the association between quintiles and brain volumes as an outcome. The models were adjusted for age, sex, education, smoking, BMI, diabetes, cardiovascular diseases, ApoE4 and HDL. P values adjusted by using conventional Bonferroni correction ($p < 0.004$) were used.

© 2019 Zhu H et al. *JAMA Network Open.*

eFigure 1. Accumulative Alzheimer Disease Incidence Rates Based on Different Cutoffs for Plasma Amylin Concentration

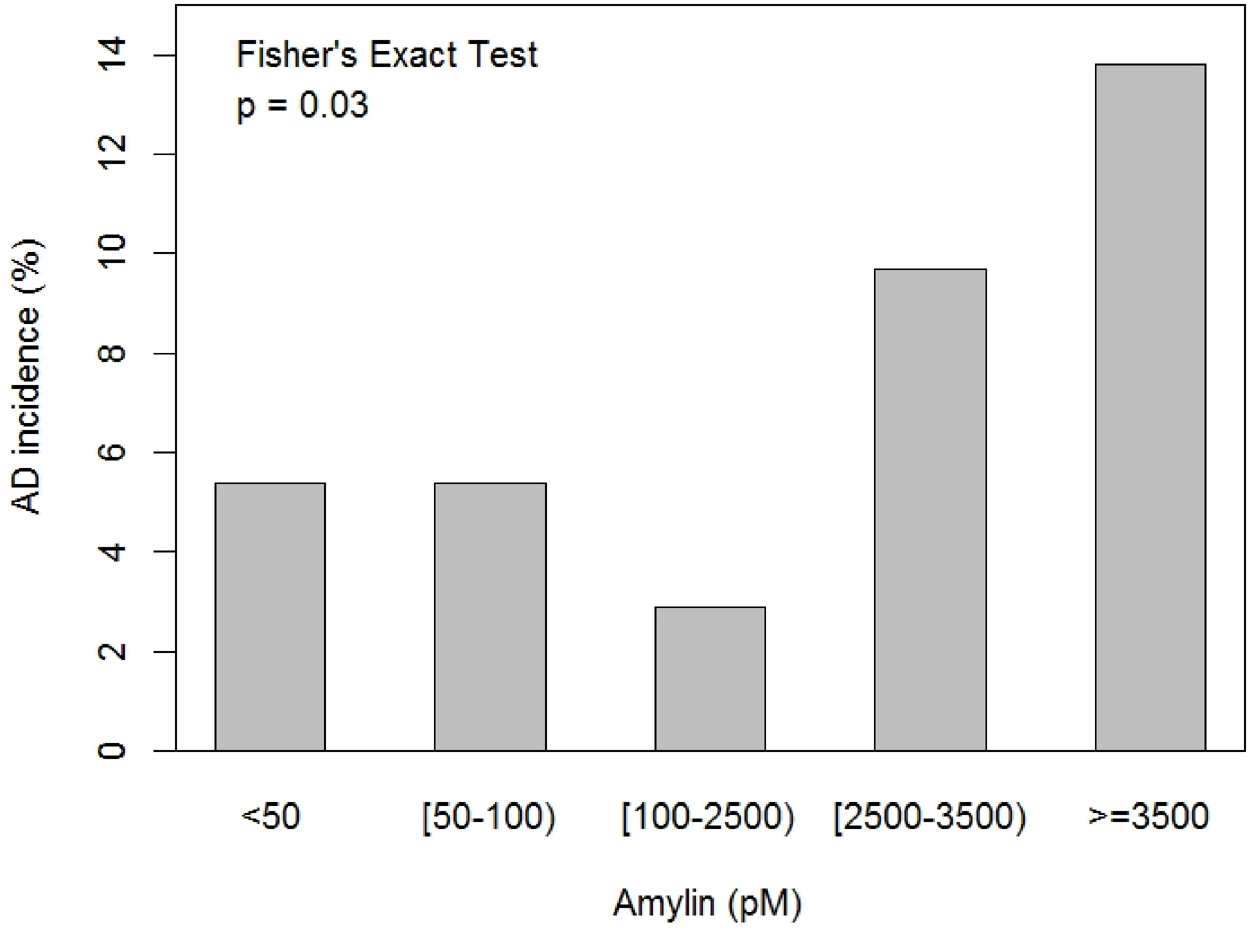

Participants were divided into five subgroups: 1) < 50 (n = 2642), 2) 50-100 (n = 185), 3) 100-2500 (n = 138), 4) 2500-3500 (n = 31) and 5) ≥ 3500 (n = 65) pM. The AD incident rates among the five plasma amylin subgroups were compared by using Fisher's Exact Test (Y-axis) with a p value illustrated.

© 2019 Zhu H et al. *JAMA Network Open.*

eFigure 2. Distribution of Plasma Amylin Concentrations Among the Population of the Framingham Heart Study

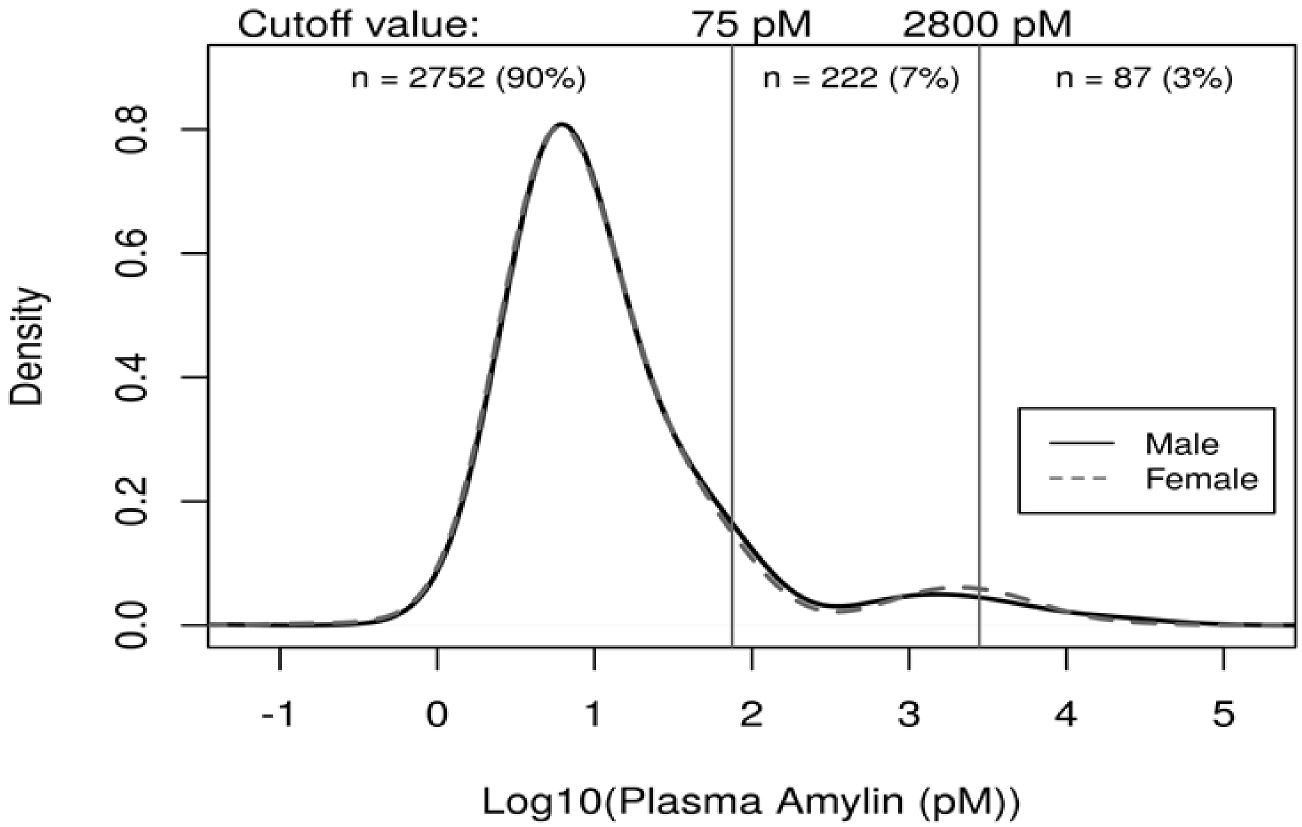

eFigure 2: Distribution of plasma amylin in the population of Framingham Heart Study
The data of plasma amylin were logarithmized (X-axis) and the distributions of particiapnts (numbers, Y-axis) in females and males were presented. The corresponding raw concentrations of plasma amylin for the cut-offs were also illustrated.

© 2019 Zhu H et al. *JAMA Network Open.*
